# Association of intraindividual tacrolimus variability with de novo donor-specific HLA antibody development and allograft rejection in pediatric kidney transplant recipients with low immunological risk

**DOI:** 10.1007/s00467-022-05426-3

**Published:** 2022-02-15

**Authors:** Maral Baghai Arassi, Laura Gauche, Jeremy Schmidt, Britta Höcker, Susanne Rieger, Caner Süsal, Burkhard Tönshoff, Alexander Fichtner

**Affiliations:** 1 Department of Pediatrics I, University Children’s Hospital Heidelberg, Heidelberg, Germany; 2 Structural and Computational Biology Unit, European Molecular Biology Laboratory, Heidelberg, Germany; 3 Institute of Immunology, Transplantation Immunology, University Hospital Heidelberg, Heidelberg, Germany

**Keywords:** Tacrolimus intra-patient variability (TacIPV), De novo donor-specific HLA antibody (*dn*DSA), Allograft rejection, Kidney transplantation (KTx), Pediatrics

## Abstract

**Background:**

Tacrolimus (Tac) intraindividual variability (TacIPV) in pediatric kidney transplant patients is only poorly understood. We investigated the impact of TacIPV on de novo donor-specific HLA antibodies (*dn*DSA) development and allograft rejection in Caucasian pediatric recipients of a living or deceased donor kidney with low immunological risk.

**Methods:**

This was a single-center retrospective study including 48 pediatric kidney transplant recipients. TacIPV was calculated based on coefficient of variation (CV%) 6–12 months posttransplant. TacIPV cutoff was set at the median (25%). Outcome parameters were *dn*DSA development and rejection episodes.

**Results:**

In total, 566 Tac levels were measured with median 11.0 (6.0–17.0) measurements per patient. The cutoff of 25% corresponded to the median CV% in our study cohort (25%, IQR 18–35%) and was comparable to cutoffs determined by receiver operating characteristic (ROC) curve analysis. High TacIPV was associated with higher risk of *dn*DSA development (HR 3.4, 95% CI 1.0–11.1, *P* = 0.047; Kaplan–Meier analysis *P* = 0.018) and any kind of rejection episodes (HR 4.1, 95% CI 1.1–14.8, *P* = 0.033; Kaplan–Meier analysis *P* = 0.010). There was a clear trend towards higher TacIPV below the age of 6 years. TacIPV (CV%) was stable over time. A TacIPV (CV%) cutoff of 30% or IPV quantification by mean absolute deviation (MAD) showed comparable results.

**Conclusions:**

High TacIPV is associated with an increased risk of *dn*DSA development and rejection episodes > year 1 posttransplant even in patients with low immunological risk profile. Therefore, in patients with high TacIPV, potential causes should be addressed, and if not resolved, changes in immunosuppressive therapy should be considered.

**Graphical abstract:**

A higher resolution version of the Graphical abstract is available as Supplementary information.

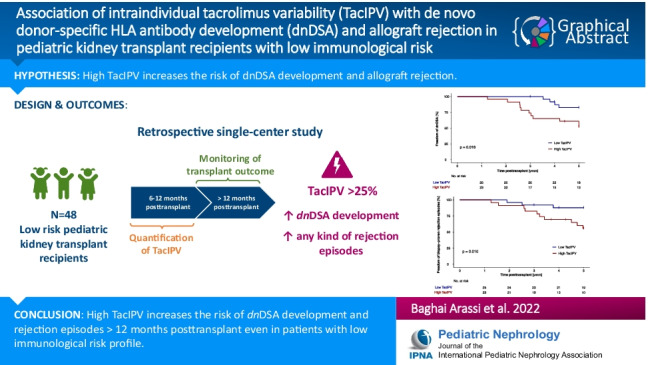

**Supplementary Information:**

The online version contains supplementary material available at 10.1007/s00467-022-05426-3.

## Introduction

Kidney transplantation (KTx) is the treatment of choice for pediatric patients with chronic kidney disease stage 5. The introduction of potent immunosuppressants such as the calcineurin inhibitor (CNI) tacrolimus (Tac) and the anti-metabolite mycophenolate mofetil (MMF) as well as anti-infective prophylaxis have substantially improved short-term transplant outcome. However, long-term graft survival remains limited [1]. While previously CNI-induced nephrotoxicity was considered the predominant reason for late graft function deterioration over time, growing evidence suggests that the causes are multifactorial and complex [2]. In this context, alloantigen-dependent factors, more specifically, de novo donor-specific antibodies (*dn*DSA) directed against human leukocyte antigens (HLA) play a major role [3]. Development of *dn*DSA is strongly associated with antibody-mediated rejection (ABMR) [4], one of the most common causes of late kidney allograft failure [5]. The exact mechanisms promoting *dn*DSA development are not yet fully understood; however, high Tac intra-patient variability (TacIPV) has recently been described as a potential contributing factor [6].

Tac is the cornerstone of modern immunosuppressive regimens. However, its narrow therapeutic index and high inter- and intraindividual variability represent a major challenge [7]. TacIPV describes fluctuations of a patient’s Tac blood trough levels over time and is impacted by multiple factors including diet, diarrhea, genetics, drug–drug interactions, drug formulation, and non-adherence [8]. Although a causal link is still missing, it is generally assumed that variable Tac exposure results in periodic under- or overexposure contributing to immune activation and nephrotoxicity, both of which negatively impact graft function [8].

In the context of adult KTx, the association between TacIPV, poor graft survival, and alloimmunity has been widely established [9–13]. In the pediatric transplant population, however, this link is less well described. Few retrospective studies demonstrated a correlation between TacIPV and alloimmunity in pediatric kidney transplant patients, but they mainly focused on either non-Caucasians, living kidney donor recipients, or patients with high immunological risk profile [14–17]. Therefore, the impact of TacIPV in Caucasian pediatric kidney transplant patients with a low immunological risk profile remains poorly understood. Furthermore, this patient population is heterogenous with regard to age, body size, and biological and psychosocial maturation, all factors which could influence TacIPV. We therefore investigated in this retrospective cohort study the association between TacIPV, *dn*DSA, rejection episodes, and graft function deterioration in pediatric kidney transplant recipients with a low immunological risk profile and analyzed whether TacIPV is associated with age.

## Patients and methods

### Study design and patient population

We conducted a retrospective observational single-center cohort study including 48 pediatric kidney transplant patients. All patients underwent kidney transplantation in the University Hospital Heidelberg, Germany, during 2006–2016. All included patients were considered as having a low immunological risk. Low immunological risk was defined as recipients of living-related donor kidney and/or at least 3 HLA matches of which at least 1 was an HLA-DR match. Patients with re-transplantation, multi-organ transplantation, positive crossmatch, less than 3 HLA matches, less than 1 HLA-DR match, and/or ABO-incompatible kidney transplantation were excluded. Other inclusion criteria were (i) HLA-DSA negativity pretransplant and within the first year posttransplant, (ii) absence of a biopsy-proven rejection between month 6 and 12 posttransplant, (iii) at least three outpatient Tac trough level measurements between month 6 and 12 posttransplant, and (iv) a tacrolimus-based immunosuppressive regimen throughout the whole follow-up period. Patients were followed-up for up to 5 years posttransplant.

Standard immunosuppressive therapy at our study center consisted of a triple immunosuppressive regimen with Tac, MMF, and methylprednisolone; the majority of patients (77%) did not receive induction therapy. Target immunosuppressive blood trough levels for Tac were slowly tapered from 10–12 µg/L in the first month to 5–8 µg/L after 6 months posttransplant. If given with everolimus (*n* = 4), target immunosuppressive blood trough levels for Tac were the following: 5–8 µg/L in the first month posttransplant, 4–7 µg/L until month 4, 3–5 µg/L thereafter. The initial MMF dose was 1200 mg/m^2^ body surface area per day, after 2 weeks posttransplant 600–900 mg/m^2^ per day, adjusted to achieve an MPA-AUC of > 40 mg*h/L. Methylprednisolone therapy was tapered to 3 mg/m^2^ (max. 5 mg/day) after 2 months posttransplant.

### Data collection

Patient demographic and baseline transplantation characteristics were collected from medical charts. In addition, data on Tac whole-blood trough levels were collected from our electronic hospital laboratory information system. Only outpatient Tac levels measured between month 6 and 12 posttransplant were included. In case of concentrations < 3 µg/L or > 20 µg/L, patient charts were manually reviewed. Erroneously high trough levels resulting from drug intake before blood sampling were excluded. TacIPV was expressed as percentage and calculated as coefficient of variation (CV%) based on the formula TacIPV = CV% = (standard deviation / mean) × 100. Unless otherwise stated, TacIPV is used synonymously with Tac CV% for the purpose of this study.

To compare TacIPV quantified by CV% to other statistical measures of TacIPV, we additionally quantified the Tac mean absolute deviation (MAD) based on the formula MAD% = {[(*X*_mean_ − *X*_1_) + (*X*_mean_ − *X*_2_) … + (*X*_mean_ − *X*_n_)] ÷ *n*}÷ *X*_mean_, where *X* is the Tac blood trough level.

Assessment of *dn*DSA was done at least yearly and during each episode of graft function deterioration and before initiation of anti-rejection therapy using LABScreen Luminex kits of One Lambda (Canoga Park, CA, USA), which use single HLA-antigen-coated beads and enable identification of immunoglobulin G (IgG) alloantibody specificities against HLA-A, -B, -C, -Cw, -DR, -DQ, and -DP antigens, as previously described [18–20]. A mean fluorescence intensity (MFI) cutoff value of > 500 was considered positive. The cutoff of 500 MFI is based on a Collaborative Transplant Study (CTS) analysis of HLA antibody cutoffs in patients with and without graft failure, which was performed in the Department of Transplantation Immunology Heidelberg. In this large multicenter study, the incidence of weak *dn*DSA or non-DSA at a mean fluorescence intensity of 500 or higher was found to be higher in the graft loss than in the non-rejector group (76 vs. 40%, *P* < 0.001) [19]. In addition, kidney transplant function was assessed by the estimated glomerular filtration rate (eGFR) (modified Schwartz formula if < 18 years or Modification of Diet in Renal Disease (MDRD) formula if ≥ 18 years) at least every 3–6 months. Biopsy-proven rejection episodes were monitored throughout the entire follow-up period and graded using the Banff 2009–15 criteria [21]. Kidney biopsies were carried out as indication biopsies due to an increase of serum creatinine (> 20% above baseline without an alternative explanation) and/or de novo persistent proteinuria > 100 g protein/mol creatinine. No protocol biopsies were performed. No patient was treated for rejection without biopsy-proven diagnosis of rejection.

### Study outcome

Primary study outcome was the association between TacIPV (months 6 to 12) and the development of *dn*DSA > year 1 posttransplant. Secondary outcome criteria were the association between TacIPV and (i) biopsy-proven rejection episodes, and (ii) graft function deterioration defined as > 50% decrease in eGFR compared to baseline (value at 4 weeks posttransplant) and/or eGFR < 30 mL/min per 1.73 m^2^. In addition, we investigated the distribution of TacIPV across three different age groups (2–5, 6–11, 12–21 years).

### Statistical analysis

Data are expressed as mean ± SD unless otherwise stated. All analyses were performed using IBM SPSS Statistics version 27 (IBM, Corp, Armonk, NY). Differences between groups were assessed using either paired *t*-test, one-way analysis of variance, Kruskall–Wallis test, Mann–Whitney *U*-test, or chi-square test as appropriate. Bonferroni correction was used for post hoc analysis. A two-sided *P* value < 0.05 was considered statistically significant. Recipients were divided into two groups, one low TacIPV and one high TacIPV group, according to the CV% cutoff of 25% corresponding to the median of TacIPV in the entire cohort. Kaplan–Meier survival analysis was performed to calculate the time to event from transplantation. For comparison of different groups, the log rank test was used. To examine risk factors for *dn*DSA development, univariate and multivariable Cox regression analyses were performed. Factors included in the final multivariable model with forward selection were donor type (living or deceased donor), steroid-free therapy one year posttransplant, high TacIPV, and cold ischemia time. Receiver operating characteristic (ROC) plots for CV% and MAD were generated, and area under the curve (AUC) and 95% confidence interval (CI) limits were calculated using the method of Hanley and McNeil [22]. Optimum cutoff level for Tac CV% was defined by the Youden Index. Biopsy-proven rejection episodes were analyzed as the pooled outcome criterion “any kind of rejection episode,” including ABMR, T-cell-mediated rejection (TCMR), and borderline rejections, because the respective number of different rejection categories was too low for a meaningful analysis.

## Results

### Clinical and demographic characteristics

A total of 48 patients, 24 girls (50.0%) and 24 boys (50.0%), undergoing kidney transplantation between 2006 and 2016 were included. Median age at transplantation was 11.3 (5.6–15.7) years. All patients were of Caucasian descent. In the majority of patients, the primary kidney disease was congenital anomalies of the kidney and urinary tract (CAKUT, 47.9%), followed by hereditary cystic diseases (14.6%) and glomerular diseases (12.5%). Twenty-one (43.8%) patients received a kidney from a living donor. All patients received Tac, MMF, and methylprednisolone as initial immunosuppressive regimen. Eight patients (16.7%) received additional induction therapy with basiliximab and 3 patients (6.3%) were administered daclizumab. The average follow-up time was 57 ± 5 months posttransplant.

We collected a total of 566 outpatient Tac trough level values with a median of 11.0 (6.0–17.0) measurements per patient. The mean Tac trough level was 6.6 ± 1.2, and the median TacIPV was 25% (IQR 18–35%). The cutoff value for high TacIPV was set at 25% corresponding to the median TacIPV in our study cohort. Baseline clinical and demographic characteristics for the high and low TacIPV cohort are shown in Table 1. There were no significant differences in baseline clinical and demographic characteristics (age, gender, HLA-matching, induction therapy, number of Tac trough levels, follow-up time) between the two cohorts. However, the low TacIPV cohort included significantly more living donor kidney transplant recipients (*P* = 0.018) and associated with that a significantly shorter cold ischemia time (*P* = 0.016).

**Table 1:** Baseline clinical and demographic characteristics of transplant patients stratified by a TacIPV cutoff value (CV%) of 25%

Baseline characteristics	Entire cohort	Low TacIPV	High TacIPV	*P* value
	*N* = 48	*N* = 25	*N* = 23	
Cold ischemia time (hrs), mean ± SD	10.0 ± 7.1	7.6 ± 5.8	12.5 ± 7.6	0.016*
Living donor, *n* (%)	21 (43.8)	15 (60.0)	6 (26.1)	0.018*
Pre-emptive donation, *n* (%)	14 (29.2)	11 (44.0)	3 (13.0)	0.018*
Recipient age, median (IQR)	11.3 (5.6–15.7)	12.6 (6.5–15.7)	9.9 (4.6–16.0)	0.556
Female recipient, *n* (%)	24 (50.0)	14 (56.0)	10 (43.5)	0.386
Primary kidney disease, *n* (%)				0.853
CAKUT	23 (47.9)	13 (52.0)	10 (43.5)	
Glomerular disease	6 (12.5)	3 (12.0)	3 (13.0)	
Cystic disease	7 (14.6)	4 (16.0)	3 (13.0)	
Other	12 (25.0)	5 (20.0)	7 (30.4)	
Number of HLA mismatches, mean ± SD				
A/B/DR	2.02 ± 1.02	1.96 ± 1.10	2.09 ± 0.95	0.672
A	0.58 ± 0.58	0.60 ± 0.58	0.57 ± 0.59	0.837
B	0.85 ± 0.55	0.80 ± 0.58	0.91 ± 0.52	0.479
DR	0.58 ± 0.50	0.56 ± 0.51	0.61 ± 0.50	0.739
Induction therapy, *n* (%)	11 (2.1)	7 (28.0)	4 (17.4)	0.431
Immunosuppressive therapy at 1 year, *n* (%)				
Everolimus	4 (8.3)	3 (12.0)	1 (4.4)	0.338
Azathioprine	3 (6.3)	0 (0)	3 (13.0)	0.062
MMF	39 (81.3)	22 (88.0)	17 (73.9)	0.212
Steroids	43 (89.6)	24 (96.0)	19 (82.6)	0.129
Tacrolimus	48 (100)	25 (100)	23 (100)	
Number of tacrolimus trough levels month 6–12 posttransplant per patient, median (IQR)	11.0 (6.0–17.0)	10.0 (5.5–17.0)	11.0 (7.0–17.0)	0.501
Tacrolimus trough level 6–12 months posttransplant, mean ± SD	6.6 ± 1.2	6.5 ± 1.2	6.8 ± 1.2	0.336
Follow-up time (months), mean ± SD	57 ± 5	57 ± 6	57 ± 5	0.779

*TacIPV*, tacrolimus intra-patient variability; *hrs*, hours; *n*, number of patients; *SD*, standard deviation; *IQR*, interquartile range; *CAKUT*, congenital anomalies of kidney and urinary tract; *HLA*, human leukocyte antigen, * *P* < 0.05

Bold print and an asterix for *P* values indicate a significant *P* value defined as *P*< 0.05

### TacIPV and *dn*DSA

Fifteen patients (31.3%) developed *dn*DSA at a median of 37 (IQR 31–50) months posttransplant. Table 2 shows the characteristics of the *dn*DSA in the immunoglobulin G single-bead antibody (IgG-SAB) test. Supplementary Table 1 shows the characteristics of donor-specific HLA antibodies in the immunoglobulin G single-bead antibody (IgG-SAB) test stratified according to TacIPV status. The *dn*DSA-positive cohort had a significantly longer cold ischemia time (*P* = 0.002) than the *dn*DSA-negative cohort which was associated with the higher number of deceased kidney donor recipients in the *dn*DSA-positive group. In addition, the *dn*DSA-positive cohort had a significantly lower number of patients with steroid therapy at year 1 posttransplant (*P* = 0.013). Other baseline clinical and demographic characteristics between patients developing *dn*DSA and those without did not differ significantly (Table 3).

**Table 2:** Characteristics of donor-specific HLA antibodies in the immunoglobulin G single-bead antibody (IgG-SAB) test

Characteristics	*dn*DSA-positive cohort (*N* = 15)
Only class I, *n* (%)	2 (13.3)
Only class II, *n* (%)	8 (53.3)
Class I and II, *n* (%)	5 (33.3)
MFI class I (median, IQR)	1486 (769–2981)
MFI class II (median, IQR)	2217 (1262–9607)
Number DQ ≥ 1, *n* (%)	9 (60)
Number DR ≥ 1, *n* (%)	5 (33.3)
Number DP ≥ 1, *n* (%)	3 (20.0)
Number A ≥ 1, *n* (%)	3 (20.0)
Number B ≥ 1, *n* (%)	2 (13.3)
Number Cw ≥ 1, *n* (%)	4 (26.7)
Number C ≥ 1, *n* (%)	1 (6.7)
Number of loci with at least one *dn*DSA per patient (median, IQR)	1 (1–3)

*dnDSA*, de novo donor-specific HLA antibodies; *class I*, HLA class I; *class II*, HLA class II; *Number*, number of DSA directed against different HLA antigens; *MFI*, mean fluorescence intensity

**Table 3:** Baseline clinical and demographic characteristics of transplant patients stratified by *dn*DSA development

Baseline characteristics	*dn*DSA positive	*dn*DSA negative	*P* value
	*N* = 15	*N* = 33	
Cold ischemia time (hrs), mean ± SD	14.4 ± 7.2	7.9 ± 6.1	0.002*
Living donor, *n* (%)	4 (26.7)	17 (51.5)	0.108
Pre-emptive transplantation, *n* (%)	2 (13.3)	12 (36.4)	0.104
Recipient age, median (IQR)	10.7 (4.1–14.1)	12.6 (6.0–16.4)	0.519
Female recipient, *n* (%)	6 (40.0)	18 (54.5)	0.350
Primary kidney disease, *n* (%)			0.211
CAKUT	5 (33.3)	18 (54.5)	
Glomerular disease	4 (26.7)	2 (6.1)	
Cystic disease	2 (13.3)	5 (15.2)	
Other	4 (27.7)	8 (24.2)	
Number of HLA mismatches, mean ± SD			
A/B/DR	2.33 ± 0.49	1.88 ± 1.17	0.155
A	0.80 ± 0.68	0.48 ± 0.51	0.079
B	0.87 ± 0.35	0.85 ± 0.62	0.916
DR	0.67 ± 0.49	0.55 ± 0.51	0.441
Induction therapy, *n* (%)	4 (26.7)	7 (21.1)	0.665
Immunosuppression at year 1, *n* (%)			
Everolimus	0 (0)	4 (12.1)	0.159
Azathioprine	2 (13.3)	1 (3.0)	0.172
MMF	11 (73.3)	28 (84.9)	0.343
Steroids	11 (73.3)	32 (97.0)	0.013*
Number of tacrolimus trough levels per patient in months 6–12 posttransplant, median (IQR)	13.0 (8.0–18.0)	9.0 (6.0–16.5)	0.208
Tacrolimus trough level in months 6–12 posttransplant, mean ± SD	6.7 ± 1.1	6.6 ± 1.3	0.868
Follow-up time (months), mean ± SD	56 ± 6	57 ± 5	0.474

*dnDSA*, de novo donor-specific antibodies; *n*, number of patients; *hrs*, hours; *SD*, standard deviation; *IQR*, interquartile range; *CAKUT*, congenital anomalies of kidney and urinary tract; *HLA*, human leukocyte antigen, * *P* < 0.05

**Fig. 1:**
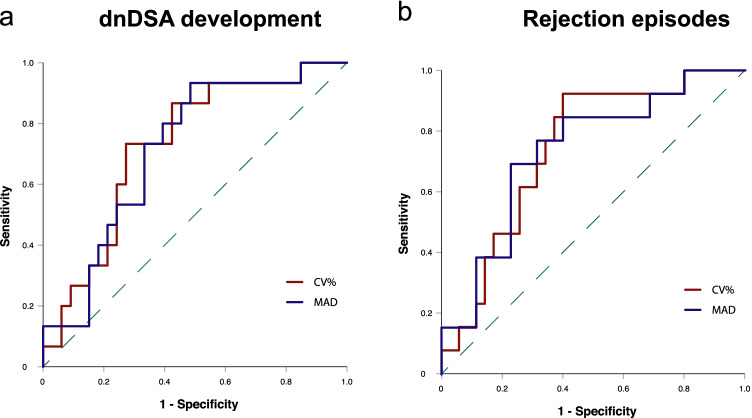
ROC curve analysis to determine the optimal TacIPV cutoff value differentiating between patients with increased alloimmune response defined as either *dn*DSA development (**a**) or biopsy-proven rejection episodes (**b**) and those without. TacIPV is quantified by coefficient of variation (CV%, red line) and mean absolute deviation (MAD, blue line)

Our cutoff value of 25% was comparable to the optimal TacIPV cutoff value determined by ROC curve analysis (Youden Index 26%), for which an area under the ROC curve value (AUROC) of 0.727 (95% CI 0.579–0.876; *P* = 0.012) indicated a moderate capacity to discriminate between patients developing *dn*DSA and those without (Fig. 1). Quantifying TacIPV based on Tac MAD did not show any additional discriminatory benefit (AUROC 0.715; 95% CI 0.565–0.865; *P* = 0.018).

**Fig. 2:**
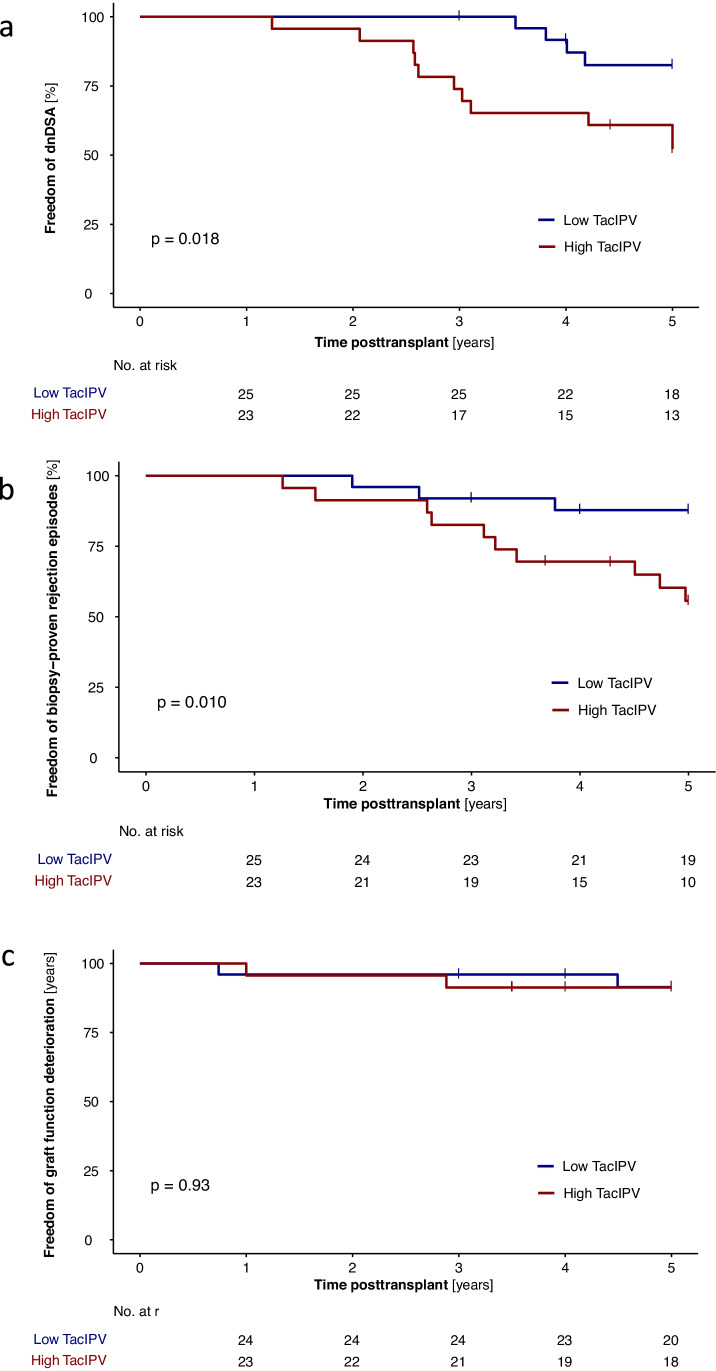
Association of tacrolimus intra-patient variability (TacIPV) with de novo donor-specific HLA antibody development (*dn*DSA) (**a**), biopsy-proven rejection episodes (**b**), and graft function deterioration (**c**) defined as > 50% decrease in eGFR compared to baseline and/or eGFR < 30 mL/min per 1.73 m^2^ beyond the first year posttransplant. Log rank *P* values of Kaplan–Meier analysis are shown

Based on the TacIPV cutoff value of 25%, Kaplan–Meier analysis revealed a strong association of high TacIPV with *dn*DSA development after the first year posttransplant (*P* = 0.018, Fig. 2). Multivariable Cox regression analysis revealed a hazard ratio of 3.4 (95% CI 1.0–11.1, *P* = 0.047, Table 4) for *dn*DSA development. When we repeated this calculation with an alternative TacIPV cutoff value of 30%, this association remained significant (Kaplan–Meier analysis *P* = 0.034, multivariable Cox regression *P* = 0.043).

**Table 4:** Risk factor analysis for de novo donor-specific HLA antibody (*dn*DSA) development

Risk factor	Unadjusted HR (95% CI)	*P* value	Adjusted HR (95% CI)	*P* value
High TacIPV (%)	3.6 (1.2–11.4)	0.028*	3.4 (1.0–11.1)	0.047*
HLA-DR mismatches		0.394*		
Cold ischemia time (hrs)^#^	5.1 (1.1–25.0)	0.042*	3.9 (1.1–14.2)	0.038*
Steroid-free therapy month 12 posttransplant	5.9 (1.9–18.8)	0.003*	8.5 (2.3–31.6)	0.001*
Donor type (living)	0.4 (0.1–1.1)	0.08		
Recipient age (years)	1.0 (0.9–1.1)	0.702		
Induction therapy	1.6 (0.5–5.2)	0.416		
Mean Tac trough concentration (µg/L)	1.0 (0.7–1.6)	0.881		

*HR*, hazard ratio; *CI*, confidence interval; *TacIPV*, tacrolimus intra-patient variability quantified as Tac coefficient of variation (CV%); *HLA*, human leukocyte antigen; * *P* < 0.05; ^#^ log transformed because nonparametric with right screw

### Association of TacIPV with rejection episodes and graft function deterioration

Late rejection episodes beyond the first year posttransplant occurred in 13 transplant recipients (27.1%). Median time to rejection episode was 37 months (IQR 27–50). Rejection types consisted of chronic active ABMR (*N* = 3 (23.1%)), acute TCMR (*N* = 2 (15.4%)), borderline rejection (*N* = 6 (46.2)), and chronic TCMR (*N* = 2 (15.4)). High TacIPV was significantly associated with biopsy-proven rejection episodes beyond the first year posttransplant (Kaplan–Meier analysis *P* = 0.010, Fig. 2). Cox regression analysis revealed a hazard ratio of 4.1 (95% CI 1.1–14.8, *P* = 0.033). Although a clear trend was visible, the association did not remain significant with an alternative TacIPV cutoff value of 30% (Kaplan–Meier analysis *P* = 0.058, multivariable Cox regression *P* = 0.069). Our cutoff value of 25% was comparable to the optimal TacIPV cutoff value determined by ROC curve analysis (Youden Index 24%) with an AUROC of 0.741 (95% CI 0.593–0.888; *P* = 0.011, Fig. 1). TacIPV quantification based on Tac MAD showed a comparable discriminatory power between patients developing any kind of rejection episodes and those who did not (AUROC 0.734; 95% CI 0.577–0.891; *P* = 0.013).

There was no significant association between TacIPV and graft function deterioration (Fig. 2). No graft losses or deaths occurred in this study population.

### TacIPV and patient age

**Fig. 3:**
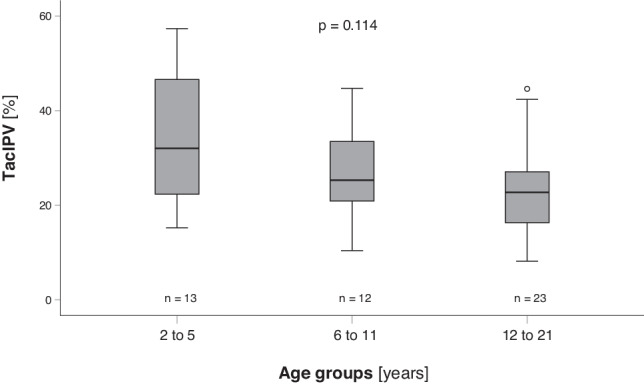
Distribution of tacrolimus intra-patient variability (TacIPV) measured as coefficient of variation (CV%) across three different age groups (2–5, 6–11, 12–21 years)

To address age heterogeneity of the pediatric patient population, we stratified our study cohort into 3 different age groups (2–5 years, 6–12 years, and > 12 years). Although not statistically significant, we detected a clear trend towards a higher TacIPV with younger age, especially after removal of one extreme value in the oldest age group (*P* = 0.114 before, *P* = 0.074 after extreme value removal, Fig. 3).

### Stability of TacIPV over time posttransplant

As a significant number of transplant-associated complications occurred ≥ 24 months posttransplant, we evaluated whether a high TacIPV between months 6 and 12 posttransplant translated into high TacIPV at later timepoints. In fact, CV% values in the time intervals 6–12 months (median 0.25), 13–18 months (median 0.26), and 19–24 months (median 0.28) posttransplant did not differ significantly (*p* = 0.831).

## Discussion

In this study, we demonstrated that an increased TacIPV is significantly associated with the development of *dn*DSA and allograft rejection in a Caucasian pediatric kidney transplant cohort with low immunological risk profile. TacIPV can be quantified by several statistical measures, and standardization is still missing. Each approach has a different set of advantages and disadvantages; however, the Tac CV% is one of the most widely used approaches, especially in the pediatric transplant population [14–16, 23–25]. In our study, we did not detect any difference in discriminatory power for development of *dn*DSA and allograft rejection between Tac CV% and Tac MAD. However, large prospective studies systematically assessing both quantification methods are needed to allow a thorough comparison of both methods.

There is no benchmark yet regarding which time period is best for TacIPV calculation. As the early phase posttransplant is characterized by high fluctuations in Tac blood levels and knowledge on the significance of TacIPV beyond 12 months after transplantation is limited, we focused on the time period between 6 and 12 months posttransplant [8, 24]. However, we could demonstrate that TacIPV is stable over time posttransplant, as high CV% between months 6 and 12 posttransplant translated into high CV% during the later posttransplant period. To our knowledge, this is the first study, in children and adults alike, showing that TacIPV is stable over time posttransplant, supporting the notion that TacIPV quantified between months 6 and 12 posttransplant could be used to predict transplant-associated events happening at later timepoints posttransplant. In fact, current literature indicates that *dn*DSA development, likely resulting from inadequate immunosuppression, is responsible for a more indolent and slowly progressive form of allograft injury, in which chronic histological changes can take long periods of time before allograft function is visibly impaired [4].

Defining a clear cutoff value to determine high TacIPV is also currently debated. Table 5 offers an overview of reported TacIPV cutoffs determined by Tac CV% in pediatric and adult kidney transplant studies. Most studies suggested a TacIPV cutoff of 30% for dividing patients into a high and low TacIPV cohort. Cutoffs were chosen empirically either based on the distribution of TacIPV values (i.e., quartiles, tertiles, or median) or based on ROC curve analyses. As in our study population, the median TacIPV was 25% and ROC curve analyses detected a similar optimal cutoff, we set the cutoff for high TacIPV at 25%. As this cutoff is relatively low compared to other studies, we additionally investigated a higher cutoff of 30%, which did not change the statistically significant association of TacIPV with *dn*DSA development. However, most likely due to our low sample size and event number, we could only detect a strong trend towards an association of TacIPV with rejection episode development when choosing a higher CV% of 30.

**Table 5:** Reported TacIPV cutoffs measured by coefficient of variation (CV%) starting at least ≥ 3 months posttransplant in adult and pediatric kidney transplant studies

Reference	TacIPV assessment period	TacIPV cutoff	Outcome parameter	Study population	*N*
Süsal et al., 2019 [26]	1, 2, and 3 years posttransplant	30%	Graft and patient survival	Adult	6638
O’Regan et al., 2016 [27]	3–12 months posttransplant	Quartiles (median) Q1: 13% Q2: 18% Q3: 25% Q4: 37%	Graft and patient survival	Adult	394
Rodrigo et al., 2016 [11]	4–12 months posttransplant	30%	Graft survival and development of *dn*DSA	Adult	310
Goodall et al., 2017 [28]	6–12 months posttransplant	Quartiles (cutoffs) Q1: < 13% Q2: ≥ 13–18% Q3: ≥ 18–25% Q4: > 25%	Graft and patient survival and rejection events	Adult	628
Vanhove et al., 2016 [13]	6–12 months posttransplant	Tertile (mean) T1: 10% T2: 18% T3: 31%	Acute and chronic histologic lesions	Adult	220
Huang et al., 2016 [29]	≤ 6 months pre-biopsy	18%	Rejection events	Adult	161
Mo et al., 2019 [30]	6–12 months posttransplant	21%	Graft loss and chronic histologic lesions	Adult	671
Solomon et al., 2019 [14]	≥ 3 months posttransplant	30%	Development of *dn*DSA and rejection events	Pediatric	38
Pizzo et al., 2016 [15]	≥ 6 months posttransplant and ≤ 6 months pre-biopsy	31%	Development of *dn*DSA and rejection events	Pediatric	23
Hsiau et al., 2011 [31]	1–12 months posttransplant	41%	Rejection events	Pediatric	46
Kaya Aksoy et al., 2019 [16]	Period 1: < 6 months posttransplant Period 2: 6–12 months posttransplant Period 3: 1 year–end of follow-up	32%	Development of *dn*DSA and graft function	Pediatric	67

*dn*DSA, de novo donor-specific antibodies; *n*, number of patients

The association between high TacIPV and graft outcome has been demonstrated in multiple studies. Borra et al. were among the first to show that high TacIPV is associated with worse kidney graft survival [9]. These findings were confirmed by several follow-up studies in adult patient cohorts revealing that high TacIPV is correlated with graft survival, rejection episodes, and *dn*DSA formation [10–13, 27, 28, 32]. In the pediatric patient cohort, Pollock-BarZiv et al. were the first to describe an association between high TacIPV and increased risk of late rejection and graft loss [17]. These findings were later validated in several other studies demonstrating a similar association of high TacIPV with late acute rejection [23, 33] and graft loss [34]. Notably, Solomon et al. linked TacIPV to *dn*DSA development in a minority cohort of mainly African American and Hispanic patients with low socioeconomic background [14]. A study by Aksoy et al. supported these findings and reported an association between high TacIPV and *dn*DSA development in living kidney donor KTx [16]. To our knowledge, our study is the first to validate these findings in a cohort of low-risk Caucasian patients including both living and deceased donor kidney transplantations. Similar to previous studies in the pediatric kidney transplant population [14–17], we detected a significant association between TacIPV and biopsy-proven rejection, underscoring the importance of TacIPV even in patients with low immunological risk profile. This association might be especially relevant for patients with relatively low Tac blood target levels as shown in our study population, where a low median Tac level combined with high TacIPV led to a considerable number of rejection episodes and *dn*DSA development even in patients with low immunological risk.

We could not detect a significant association with graft function deterioration, probably due to the overall low incidence of this event and the relatively low number of patients.

Interestingly, we saw a clear trend towards a higher TacIPV in patients below the age of 6 years. This finding is in contrast to studies suggesting a higher TacIPV in adolescents due to lower therapy adherence [31, 35, 36], but is consistent with a study by Prytula et al. [33]. Although our study does not allow any causal inferences, a possible explanation might be a generally higher susceptibility to infections, in particular gastrointestinal, at younger age, which is known to interfere with TacIPV [8] because diarrhea leads to increased Tac bioavailability. Other potential mechanisms are differences in the metabolism of Tac among pediatric age groups [37], the number of Tac dose changes, which has previously been associated in adult kidney transplant recipients with increased IPV [9, 38], age-specific differences in the frequency of infections and infection-associated inflammation [39], and/or age-specific differences in the composition of the gut microbiota which appears to contribute to Tac metabolism [40]. Future studies should therefore consider age-group stratification and detailed adherence and infection monitoring for an adequate assessment of the relationship between age and TacIPV.

Other factors besides variability in Tac exposure are likely to have contributed to the development of *dnD*SA formation. In our patient cohort, the *dn*DSA-positive subgroup had a longer cold ischemia time than the *dn*DSA-negative subgroup and less frequently received steroids at year 1 posttransplant than the *dn*DSA-negative subgroup. However, in a large prospective randomized trial on steroid avoidance in pediatric kidney transplantation, steroid-free immunosuppression was not associated with a higher frequency of *dn*DSA development [41].

Our study has several limitations. First, the investigated patient cohort is relatively small and findings may not be generalizable to highly heterogenous pediatric patient populations. Second, given the retrospective design of this study, we cannot make any statement about the causal link between TacIPV and *dn*DSA formation as information on factors known to be associated with TacIPV such as non-adherence or infectious episodes was not available. Despite these limitations, our study provides important insights into the relationship between Tac variability, *dn*DSA development, and allograft rejection in a pediatric cohort with low immunological risk profile.

In conclusion, our data indicate that high TacIPV between months 6 and 12 posttransplant is, irrespective of quantification by CV% or MAD, associated with a higher risk of developing *dn*DSA directed against HLA and allograft rejection beyond the first year posttransplant. However, further validation in large-scale, prospective trials is needed to qualify TacIPV as a predictive diagnostic marker for increased risk of alloimmunity. If accomplished, TacIPV could serve as a cheap and non-invasive biomarker for identifying patients at risk of developing *dn*DSA and long-term graft loss. For now, in patients with highly variable Tac trough blood concentrations, potential causes of high TacIPV should be addressed and if not resolved, a change in immunosuppressive therapy should be considered to prevent *dn*DSA development with the overall goal of preventing rejection and improving long-term graft outcomes.

## Supplementary Information

Below is the link to the electronic supplementary material.

Supplementary file1 (PPTX 386 KB)

Supplementary file2 (XLSX 11.3 KB)

## Abbreviations

ABMR: Antibody-mediated rejection
CNI: Calcineurin inhibitor
CV%: Coefficient of variation in percent
*dn*DSA: De novo donor-specific antibodies
eGFR: Estimated glomerular filtration rate
HLA: Human leukocyte antigen
KTx: Kidney transplantation
MAD: Mean absolute deviation
MDRD: Modification of diet in renal disease
MFI: Mean fluorescence intensity
MMF: Mycophenolate mofetil
MPA: Mycophenolic acid
Tac: Tacrolimus
TacIPV: Tacrolimus intra-patient variability
TCMR: T cell-mediated rejection
