# Supplementary material for: Association of intraindividual tacrolimus variability with de novo donor-specific HLA antibody development and allograft rejection in pediatric kidney transplant recipients with low immunological risk
Source: Pediatr Nephrol. 2022 Feb 15;37(10):2503–14. doi: 10.1007/s00467-022-05426-3 (PMC9395307; doi:10.1007/s00467-022-05426-3)
Supplement: Supplementary file 1 — Supplementary file1 (PPTX 386 KB) [file 467_2022_5426_MOESM1_ESM.pptx]

## Slide 1
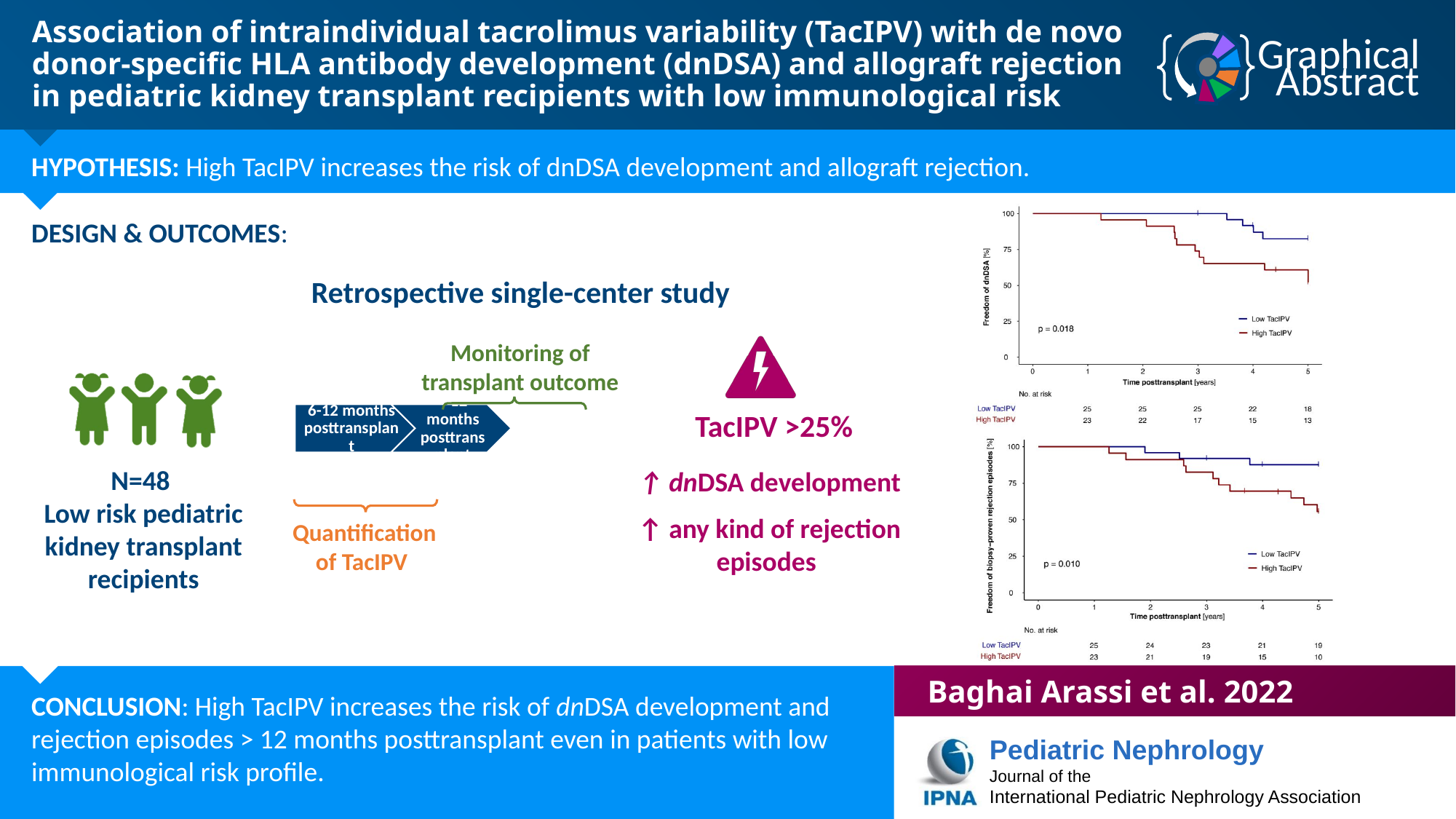

Association of intraindividual tacrolimus variability (TacIPV) with de novo donor-specific HLA antibody development (dnDSA) and allograft rejection in pediatric kidney transplant recipients with low immunological risk
HYPOTHESIS: High TacIPV increases the risk of dnDSA development and allograft rejection.
DESIGN & OUTCOMES:
Retrospective single-center study
TacIPV >25%
Monitoring of transplant outcome
Quantification of TacIPV
N=48
Low risk pediatric kidney transplant recipients
↑ dnDSA development
↑ any kind of rejection episodes
Baghai Arassi et al. 2022
CONCLUSION: High TacIPV increases the risk of dnDSA development and rejection episodes > 12 months posttransplant even in patients with low immunological risk profile.
